# COVID-19 Autopsies Reveal Underreporting of SARS-CoV-2 Infection and Scarcity of Co-infections

**DOI:** 10.3389/fmed.2022.868954

**Published:** 2022-04-14

**Authors:** Nathalie Schwab, Ronny Nienhold, Maurice Henkel, Albert Baschong, Anne Graber, Angela Frank, Nadine Mensah, Jacqueline Koike, Claudia Hernach, Melanie Sachs, Till Daun, Veronika Zsikla, Niels Willi, Tobias Junt, Kirsten D. Mertz

**Affiliations:** ^1^Institute of Pathology, Cantonal Hospital Baselland, Liestal, Switzerland; ^2^Department of Radiology, University Hospital Basel, Basel, Switzerland; ^3^Novartis Institutes for BioMedical Research, Basel, Switzerland; ^4^University of Basel, Basel, Switzerland

**Keywords:** COVID-19, SARS-CoV-2, autopsy, mortality, respiratory failure, infection, bacterial co-infection

## Abstract

Coronavirus disease 2019 (COVID-19) mortality can be estimated based on reliable mortality data. Variable testing procedures and heterogeneous disease course suggest that a substantial number of COVID-19 deaths is undetected. To address this question, we screened an unselected autopsy cohort for the presence of SARS-CoV-2 and a panel of common respiratory pathogens. Lung tissues from 62 consecutive autopsies, conducted during the first and second COVID-19 pandemic waves in Switzerland, were analyzed for bacterial, viral and fungal respiratory pathogens including SARS-CoV-2. SARS-CoV-2 was detected in 28 lungs of 62 deceased patients (45%), although only 18 patients (29%) were reported to have COVID-19 at the time of death. In 23 patients (37% of all), the clinical cause of death and/or autopsy findings together with the presence of SARS-CoV-2 suggested death due to COVID-19. Our autopsy results reveal a 16% higher SARS-CoV-2 infection rate and an 8% higher SARS-CoV-2 related mortality rate than reported by clinicians before death. The majority of SARS-CoV-2 infected patients (75%) did not suffer from respiratory co-infections, as long as they were treated with antibiotics. In the lungs of 5 patients (8% of all), SARS-CoV-2 was found, yet without typical clinical and/or autopsy findings. Our findings suggest that underreporting of COVID-19 contributes substantially to excess mortality. The small percentage of co-infections in SARS-CoV-2 positive patients who died with typical COVID-19 symptoms strongly suggests that the majority of SARS-CoV-2 infected patients died from and not with the virus.

## Introduction

The coronavirus disease 2019 (COVID-19) global pandemic is regarded as the cause of substantial excess mortality in 2020 and 2021 in Europe, as the cycles of excess mortality paralleled the waves of the pandemic (1, 2). The European Union experienced two cycles of excess mortality during the pandemic so far: The first cycle between March and May 2020 reached a 25.1% excess rate in April 2020, and the second cycle between August 2020 and February 2021 reached a 40.6% excess rate in November 2020 (2). However, there is little evidence for a causal relationship of COVID-19 and excess mortality.

One recurrent question was whether COVID-19 patients with a fatal outcome die from or with SARS-CoV-2 (3, 4). In particular, the frequency of bacterial and fungal superinfections in critically ill COVID-19 patients and their contribution to SARS-CoV-2 associated mortality remain unclear (5). Indeed, signs, symptoms, and laboratory abnormalities in patients with SARS-CoV-2 pneumonia are identical to those of community-acquired bacterial pneumonia. In addition, pneumonia caused by bacterial superinfections is a well-known complication of respiratory virus infections. For example, *Staphylococcus aureus* infection increases the morbidity and mortality of patients suffering from influenza (6–12). In addition, respiratory infections caused by other coronaviruses such as severe acute respiratory syndrome coronavirus (SARS-CoV) and Middle East respiratory syndrome coronavirus (MERS-CoV) are associated with bacterial or fungal co-infections (13–16).

Even though data about the incidence of bacterial superinfections is scarce and variable (17–19), empirical antibiotic therapy is widely used in hospitalized COVID-19 patients (20). In order to optimize the therapy of COVID-19 patients and to avoid emergence of multidrug-resistant microorganisms, it is essential to gain more knowledge about the incidence of superinfections in critically ill patients and the effectiveness of antibiotics in this setting.

Here we describe an autopsy cohort of the Institute of Pathology Liestal during the first and second waves of the COVID-19 pandemic in Switzerland, i.e., before protective vaccines became available. To determine the incidence of SARS-CoV-2 in an unselected cohort of autopsy patients, we tested all autopsies that we performed during this time period for presence of SARS-CoV-2 viral RNA. This is of particular interest because of the suspected underreporting of SARS-CoV-2 infection rates (21, 22). Indeed, we identified a high number of previously unknown COVID-19 cases *post mortem*. To evaluate the incidence of respiratory co-infections in deceased COVID-19 patients, we analyzed autoptic lung tissues of all patients for diverse respiratory pathogens (bacteria, viruses and *Pneumocystis jirovecii*) using a novel TaqMan array card (Supplementary Table 1). We found bacterial co-infections only in 25% of COVID-19 deaths. The majority of SARS-CoV-2 positive patients died with typical respiratory symptoms of COVID-19, yet without additional co-infections or malignancies.

The two main aims of this study were: (i) to investigate potential underreporting of COVID-19 deaths in Switzerland during the first and second wave of the pandemic, and (ii) to determine the frequency of patients who died with SARS-CoV-2 in their lungs and typical COVID-19 symptoms, without having been reported as COVID-19 during their lifetime. This was achieved through analysis of SARS-CoV-2 and common respiratory pathogens in lungs in a series of 62 well-annotated unselected autopsies. Our study is relevant to the understanding of COVID-19, as it suggests that a substantial number of SARS-CoV-2 positive patients with lethal outcome were not reported as COVID-19, and that most COVID-19 patients died without detectable co-infections in their lungs or other diseases. This allows to infer that most patients died from, not with, SARS-CoV2.

## Materials and Methods

### Cohort Description

All autopsies that were conducted at the Institute of Pathology Liestal, Switzerland, between 1st of March 2020 and 31st of May 2020 (first COVID-19 pandemic wave in Switzerland) and between 1st of October 2020 and 31st of January 2021 (second pandemic wave in Switzerland) were included in our study.

### Clinical Data

Medical records of all deceased patients were evaluated for comorbidities, nasopharyngeal SARS-CoV-2 swab test results within 2 weeks before death, symptoms at hospital admission and during hospitalization, duration of hospitalization, bloodstream infections, and systemic antibiotic and/or antifungal treatment (>24 h) during hospitalization. As reported by Struyf et al., COVID-typical symptoms were considered to be fever, cough, dyspnea, sore throat, fatigue, muscle aches, shiver, rhinorrhea, loss of taste and/or odor, headache and diarrhea (23). Clinicopathological details of patients for the entire cohort and stratified by COVID-19 waves in Switzerland are summarized in Table 1.

**TABLE 1:** Clinicopathological details of patients.

Characteristics	N or median	range or%
**A**		
**All patients (*n* = 62)**		
Age (years)	76.5	38–97
Sex	M 40, F 22	
BMI (kg/m^2^)	25.5	13–59
Hospitalization time (days)	4	0–50
**Major comorbidities**		
Chronic cardiovascular disease	51	82%
Hypertension	49	79%
Chronic respiratory disease	33	53%
Chronic renal disease	28	45%
Cognitive impairment	27	44%
Diabetes mellitus	24	39%
Malignoma	21	34%
**Most common lung pathological features**		
Exudative phase of diffuse alveolar damage	11	18%
Organizing phase of diffuse alveolar damage	7	11%
Acute bronchopneumonia	21	34%
**B**		
**First pandemic COVID-19 wave in Switzerland**		
Total autopsies in first wave	25	100%
Clinically known SARS-CoV-2 positive cases	9	36%
Unexpected SARS-CoV-2 positive cases	5	20%
SARS-CoV-2 negative cases	11	44%
COVID-19 patients with thromboembolic events	4	29%
COVID-19 patients with bacterial co-infections	2	14%
COVID-19 patients with antibiotics	11	79%
Average number of reported COVID-19 symptoms	3.6	0–6
**Second pandemic COVID-19 wave in Switzerland**		
Total autopsies in second wave	37	100%
Clinically known SARS-CoV-2 positive cases	9	24%
Unexpected SARS-CoV-2 positive cases	5	14%
SARS-CoV-2 negative cases	23	62%
COVID-19 patients with thromboembolic events	3	21%
COVID-19 patients with bacterial co-infections	5	36%
COVID-19 patients with antibiotics	8	57%
Average number of reported COVID-19 symptoms	2.7	0–7

***(A)** Entire autopsy cohort, **(B)** stratified by COVID-19 pandemic waves in Switzerland.*

### Autopsy Findings

The cause of death was determined by full body autopsy including comprehensive histological examinations and was categorized as death from respiratory failure, cardiovascular death or other kind of death. Malignant underlying diseases were subdivided into “not metastasized and not relevant for death,” if the diagnosis of a malignancy was confirmed to be a non-final stage and if the malignancy was not linked to the cause of death, and “final stage and relevant for death,” if the malignancy was found to be in a final stage and/or directly linked to the cause of death.

### Sample Collection

Representative samples from different areas of the lungs of each autopsy patient were immediately preserved in 4% phosphate-buffered formalin during the autopsy. Formalin fixed and paraffin embedded (FFPE) tissue sections were stained using standard histological staining protocols (hematoxylin and eosin, H&E and elastica van Gieson, EvG). All sections were screened for histological characteristics. Neutrophilic infiltration and diffuse alveolar damage (DAD) were quantified by at least two board-certified pathologists. Neutrophilic infiltration was graded as follows: no neutrophilic granulocytes detected in an H&E stained section (grade 0), few neutrophilic granulocytes (grade 1), moderate number of neutrophilic granulocytes (grade 2) and numerous neutrophilic granulocytes (grade 3). Only cases with grade 2 or 3 neutrophilic infiltration were considered to suffer from acute bronchopneumonia. DAD was graded as follows: exudative phase (grade 1), proliferative phase (grade 2) and fibrotic phase (grade 3).

### Quantification of SARS-CoV-2 in Formalin Fixed and Paraffin Embedded Tissue Samples

RNA was extracted from one representative FFPE lung tissue sample of each autopsy patient. SARS-CoV-2 was quantified and viral load was calculated as previously described, using the TaqMan 2019-nCoV Assay Kit v1 (Cat No. A47532, ThermoFisher Scientific, Waltham, MA, United States) and the TaqMan 2019-nCoV Control Kit v1 (Cat No. A47533, ThermoFisher Scientific, Waltham, MA, United States) (24). The results obtained from one representative lung tissue sample were confirmed by the same analysis on at least one other independent lung tissue sample of each case. In addition to lung tissues, SARS-CoV-2 viral load was determined in representative tissue samples of heart, thyroid, kidney, adrenal gland, pancreas, liver and spleen of each patient.

### Detection of Other Microbes Using a TaqMan Array Card Technology

Testing for respiratory pathogens was performed using an early access version of the TrueMark Respiratory Panel 2.0 (Cat No. A49047, ThermoFisher Scientific, Waltham, MA, United States). This 384 well plate allows the analysis of eight samples for 48 pre-spotted TaqMan assays, which target a total of 42 individual respiratory pathogens (Supplementary Table 1). For each sample, 80 ng of total RNA were converted to cDNA. The pre-amplified cDNA of up to eight samples was applied to the TrueMark Respiratory Panel 2.0 TaqMan array card and measured by the QuantStudio 7 Pro Real-Time PCR System (Cat No. A43165, ThermoFisher Scientific, Waltham, MA, United States). Pathogen-specific assays with a Ct value < 27 were scored as positive.

### Validation by Immunohistochemistry and *in situ* Hybridization

Immunohistochemical staining for *Pneumocystis jirovecii* was performed using a monoclonal mouse antibody (clone 3F6) from Agilent (Santa Clara, CA, United States), a BOND-III fully automated stainer and BOND kits (Leica, Wetzlar, Germany). Slides were pre-treated with enzyme 1 (AR9551) for 20 min at 25°C, stained with the antibody for 60 min at RT, and then with the BOND Polymer Refine Red Detection kit. Finally, slides were counterstained with hematoxylin for 5 min.

Epstein-Barr virus (EBV) *in situ* hybridization was performed using the BOND ready-to-use ISH EBER probe (PB0589), a BOND-III fully automated stainer and BOND kits, all from Leica. After incubation with the ISH probe for 4 h at RT, slides were stained with the BOND Polymer Refine Red Detection kit and counterstained with hematoxylin for 5 min.

### Statistical Analysis

Statistical analyses were performed using GraphPad Prism (version 6).

### Ethics Statement

This study was conducted according to the principles expressed in the Declaration of Helsinki. Ethics approval was obtained in written form from the Ethics Committee of Northwestern and Central Switzerland (Project-ID 2020-00629). For all patients, personal family consent was obtained for the autopsy and sample collection, in line with the Swiss law and the Ethics approval.

## Results

### Patient Characteristics and Autopsy Findings

**FIGURE 1:**
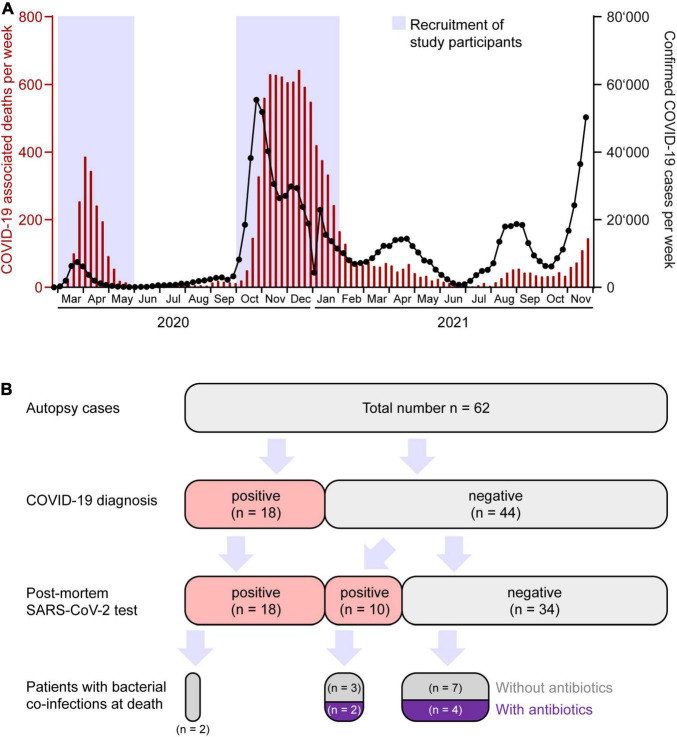
Coronavirus disease 2019 (COVID-19) epidemiology and stratification of patients. **(A)** Weekly numbers of COVID-19 cases (black interconnected dots, right axis) and deaths (red bars, left axis) in Switzerland from March 2020 until November 2021. *Blue* boxes represent time windows for collection of consecutive autopsies. Source of data: Bundesamt für Gesundheit (BAG) Switzerland, December 12, 2021. **(B)** Partition of autopsy cohort according to COVID-19 diagnosis, SARS-CoV-2 infection, co-infection, and antibacterial treatment.

We performed a total of 62 autopsies of patients assigned from regional hospitals in North Western Switzerland between 1st of March 2020 and 31st of May 2020 (*n* = 25, 1st COVID-19 wave in Switzerland) and 1st of October 2020 and 31st of January 2021 (*n* = 37, 2nd COVID-19 wave) (Figures 1A,B). Our data need to be put in perspective to the public health scenario and patient management which differed between the first and second pandemic waves in Switzerland (25–28).

**FIGURE 2:**
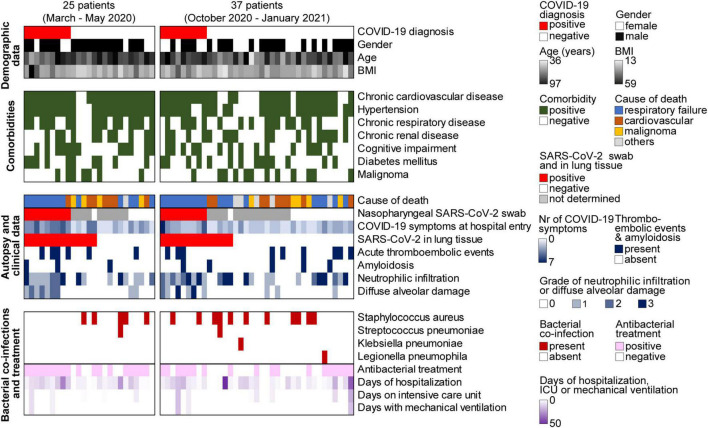
Overview of patient characteristics, clinical data, and autopsy findings of all 62 autopsies during the first (*n* = 25) and second (*n* = 37) wave of the COVID-19 pandemic. BMI, body mass index; ICU, intensive care unit.

Our autopsy cohort consisted of 65% males (*n* = 40) and 35% females (*n* = 22) with a median age of 76.5 years (range 36–97). Median body mass index (BMI) was 25.5 kg/m^2^ (13–59 kg/m^2^). None of the patients had been COVID-19 vaccinated. In Switzerland, the vaccination campaign started in early January 2021, first limited to very specific subgroups of the population (27). Consequently, vaccination did not play a role during the first and second wave of the pandemic in Switzerland. Patient characteristics, clinical data and autopsy findings of all 62 cases are summarized in Figure 2 and Table 1.

The most common comorbidities of these 62 deceased patients included chronic cardiovascular disease (*n* = 51, 82%), hypertension (*n* = 49, 79%), chronic respiratory disease (*n* = 33, 53%), chronic renal disease (*n* = 28, 45%), cognitive impairment (*n* = 27, 44%), diabetes mellitus (*n* = 24, 39%), and malignancies (*n* = 21, 34%), among those *n* = 12 in a final stage (19%) (Figure 2).

In 50% (*n* = 31) of all autopsy cases, the cause of death as determined by full body autopsy including comprehensive histological evaluation was respiratory failure due to pneumonia (*n* = 15), DAD (*n* = 10), or a combination of both (*n* = 6) (Figure 2). Three of these 31 patients were suffering from a malignant final stage tumor as an underlying condition. In 26% (*n* = 16) of all autopsy cases, the cause of death was of cardiovascular origin. Among those, five patients had an acute myocardial infarction and one patient died from an acute aortic rupture, and in 10 patients, a cardiovascular cause of death was assumed due to their clinical history of acute cardiac decompensation and autopsy findings with unequivocal cardiac pathologies (severe cardiac amyloidosis, severe cardiac hypertrophy, pulmonary edema and/or pleural effusion). In the group of patients who died from a cardiovascular cause of death, no individual had an additional malignant final stage tumor. Another 14.5% (*n* = 9) of the patients succumbed to a final stage malignancy, and the remaining 9.5% (*n* = 6) of all autopsy patients died from other causes.

### Patients With a Clinically Known Coronavirus Disease 2019 Diagnosis Prior to Autopsy

Among the 62 autopsy patients, 18 (29%) were diagnosed with COVID-19 prior to death by detection of SARS-CoV-2 in nasopharyngeal swabs (9 patients during the first, 9 patients during the second COVID-19 wave in Switzerland) (Figure 2). The majority (*n* = 14) of these 18 SARS-CoV-2 positive patients was tested once at 3–29 days before death, and some (*n* = 4) even had a second confirmatory test while still alive. The remaining 44 autopsy cases were declared COVID-19 negative (16 patients during the first, 28 patients during the second wave). Within 2 weeks prior to death, 19 (43%) out of these 44 patients received a nasopharyngeal swab test for SARS-CoV-2 with a negative result (6 patients during the first, 13 patients during the second wave). The remaining 25 (57%) out of these 44 deceased patients were not tested for the presence of SARS-CoV-2 while still alive (*n* = 10 during the first, *n* = 15 during the second wave).

At the time of hospital admission, all 18 patients with a known COVID-19 diagnosis had COVID-typical symptoms such as cough, fever, dyspnea, sore throat, fatigue, muscle ache, shiver, loss of odor, diarrhea or headache. The number of reported COVID-19 related symptoms in SARS-CoV-2 positive patients during the first wave was 3.6 on average and higher than during the second wave (average of 2.7). Thirteen (72%) out of these 18 known COVID-19 patients showed no growth of bacteria in blood culture during hospitalization. The remaining 5 did not get a blood culture test.

Autopsy findings revealed that 17 out of the 18 COVID-19 patients died from respiratory failure due to pneumonia and/or DAD. DAD was seen in the exudative phase (*n* = 6), proliferative phase (*n* = 5) and fibrotic phase (*n* = 2). One COVID-19 patient revealed no morphological alterations in his lungs and died from cardiac tamponade following subacute myocardial infarction due to a thrombotic coronary event.

Among all autopsy cases, 15 (24%) patients revealed an acute thromboembolic event such as pulmonary embolism (*n* = 6), deep vein thrombosis (*n* = 3), coronary thrombosis (*n* = 3), thrombotic microangiopathy (*n* = 3) (pulmonary, renal, and adrenal) and/or disseminated intravascular coagulation (DIC) (*n* = 3) indicating abnormally activated blood coagulation (hypercoagulability) (Figure 2). Seven (39%) out of 18 COVID-19 patients (4 patients during the first and 3 patients during the second wave) had a thromboembolic event. In contrast, a thromboembolic event was found in only 8 (18%) out of 44 non-COVID-19 patients, in line with previous observations that hypercoagulation is a typical consequence of SARS-CoV-2 (29–31).

Eight (13%) out of all autopsy cases were suffering from senile amyloidosis involving the heart and in two cases also vessels of heart and lung. Three (17%) out of 18 COVID-19 patients and 5 (11%) out of 44 non-COVID-19 patients were affected by a senile amyloidosis. This supports our previous finding in a different autopsy cohort that amyloidosis is more frequent among COVID-19 cases with a fatal outcome (31).

### Post Mortem Detection of SARS-CoV-2 in Autopsy Patients

All 62 patients of our autopsy cohort were tested *post mortem* for the presence of SARS-CoV-2 by quantitative PCR. In total, SARS-CoV-2 was detected in FFPE lung tissues of 28 (45%) cases, including 10 (16%) out of 62 autopsy patients without previously diagnosed COVID-19 (5 of 25 (20%) patients during the first and 5 of 37 (14%) patients during the second pandemic peak, Table 1). Two (20%) out of these 10 patients were even tested negative for SARS-CoV-2 *intra vitam* within 24 h before death, while the other 8 were never tested. These data suggest substantial underreporting of SARS-CoV-2 infections, either due to false negative swab tests or due to non-systematic testing.

Of the 10 unexpected SARS-CoV-2 positive patients, one died of respiratory failure with histology showing heavy inflammatory infiltrates including signs of acute bronchopneumonia (massive neutrophilic infiltration) in his lung tissues, i.e., a COVID-19 typical picture. Three had characteristic COVID-19 symptoms at hospital admission such as cough, fever, dyspnea, sore throat and/or fatigue. The autopsy of one more patient showed an acute thromboembolic coronary event with consecutive cardiovascular failure, and this patients also had COVID-19 typical symptoms *ante mortem*, compatible with a COVID-19 diagnosis. Overall, 5 unexpected SARS-CoV-2 positive patients showed clinical and/or autopsy findings consistent with COVID-19. For 5 other patients, no characteristic symptoms or autopsy findings hinted to COVID-19. These data suggest a substantial underreporting of COVID-19 related deaths and variable reported causes of death for SARS-CoV-2 infected patients. Clinicopathological details of all SARS-CoV-2 positive patients and SARS-CoV-2 negative patients are summarized in Supplementary Tables 2, 3.

**FIGURE 3:**
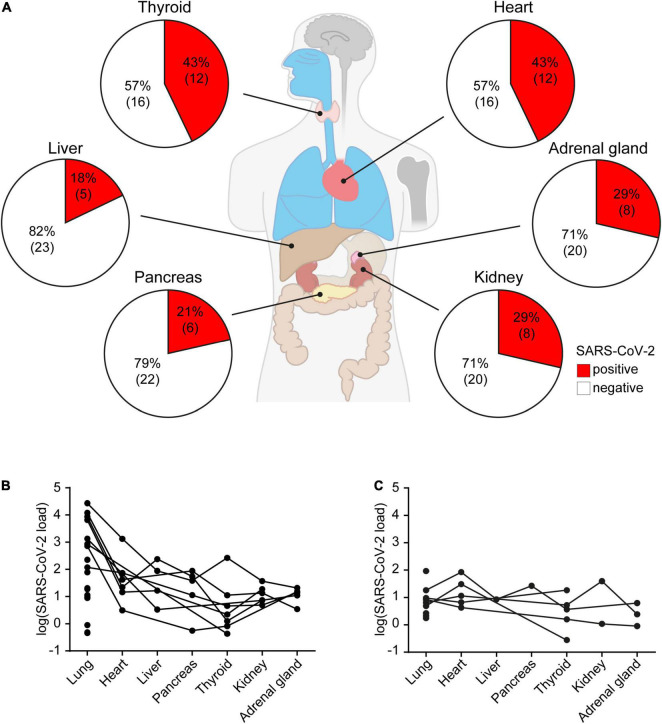
SARS-CoV-2 organ distribution in all 28 autopsy cases with SARS-CoV-2 positive lung tissues. **(A)** Percentage of SARS-CoV-2 positive heart, adrenal gland, kidney, pancreas, liver and thyroid samples of all SARS-CoV-2 infected patients. Viral load in organs of **(B)** patients with a clinically known COVID-19 diagnosis (*n* = 18), and **(C)** unexpected SARS-CoV-2 positive cases (*n* = 10). Data represent median of three technical replicates, lines connect data from the same individual. Median viral loads of SARS-CoV-2 in different organs are listed in Supplementary Table 4.

In about half (*n* = 13, 46%) of the 28 *post mortem* SARS-CoV-2 positive autopsy cases, SARS-CoV-2 was also detected outside the lung, i.e., in heart (12/28, 43%), thyroid (12/28, 43%), kidney (8/28, 29%), adrenal gland (8/28, 29%), pancreas (6/28, 21%) and liver (5/28, 18%) (Figure 3A). For the vast majority of SARS-CoV-2 positive patients, both those with a previously known COVID-19 diagnosis (Figure 3B) and those with an unexpected COVID-19 diagnosis *post mortem* (Figure 3C), highest viral copy numbers were detected in the lungs. In patients with a clinically known COVID-19 diagnosis, about fivefold more virus was detected in most organs on average, compared to the 10 patients who were unexpectedly positive for SARS-CoV-2 *post mortem* (Figures 3B,C). However, the differences in viral loads between clinically known and unexpected SARS-CoV-2 positive cases were not statistically significant in any of the organs, likely due to large variations (Supplementary Table 4).

### Post Mortem Detection of Other Respiratory Pathogens in Lung Tissues of Autopsy Patients

**FIGURE 4:**
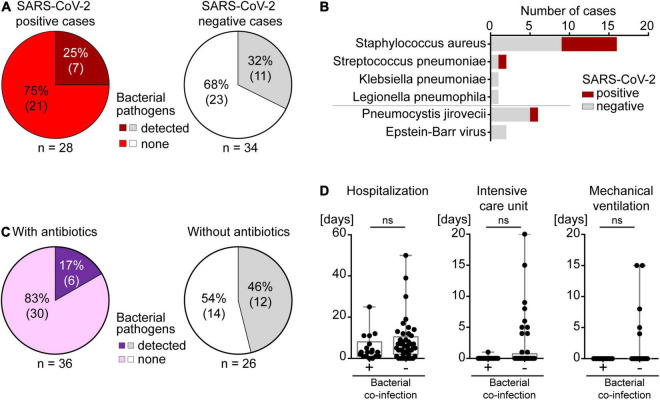
Co-infections in autopsy cases. **(A)** *Left*, SARS-CoV-2 positive and *right*, SARS-CoV-2 negative patients. **(B)** Types of co-infections identified in the autopsy cohort of SARS-CoV-2 positive and negative patients. **(C)** *Left*, bacterial co-infections in individuals with antibiotic treatment. *Right*, bacterial co-infections in individuals without antibiotic treatment. **(D)** Bacterial co-infections in relation to duration of hospitalization, ICU care or mechanical ventilation.

Lung tissues of all 62 autopsy patients were analyzed for 42 respiratory pathogens using a TaqMan array card (Supplementary Table 1). This multiplexed real-time quantitative polymerase chain reaction (qPCR) array represents the first systematic test for respiratory pathogens in formalin-fixed paraffin-embedded (FFPE) tissue samples. In all 28 autopsy cases that were identified as SARS-CoV-2 positive *post mortem* by qPCR testing (TaqMan2019-nCoV assay kit v1), SARS-CoV-2 was also detected by this respiratory TaqMan array card. SARS-CoV-2 was not detected in the 34 other autopsy patients by either of the two methods. Out of the 42 respiratory pathogens on this array card, only 4 bacterial pathogens were identified in our cohort, in 18/62 (29%) autopsy lung tissues: *Staphylococcus aureus*, *Streptococcus pneumoniae*, *Klebsiella* pneumonia and *Legionella* pneumoniae (Figures 2, 4A). The most frequently detected pathogen was *Staphylococcus aureus* (Figure 4B), which was found in 26% of SARS-CoV-2 negative patients (*n* = 9) and as a co-infection in 25% of SARS-CoV-2 positive patients (*n* = 7).

In only 2 (11%) of the 18 patients with a clinically known COVID-19 diagnosis, *Staphylococcus aureus* was detected in *post mortem* lung tissues, and histology showed acute bronchopneumonia without DAD. Both patients did not receive empirical antibiotics nor did they get blood culture testing for growth of bacteria during hospitalization. In the remaining 16 COVID-19 patients, no pathogens other than SARS-CoV-2 were detected in lung tissues, and all of these 16 patients had received prophylactic empirical antibiotics. This indicates a protective effect of empirical antibiotic therapy in COVID-19 patients (Figure 4C). In the 10 patients who were unexpectedly positive for SARS-CoV-2 *post mortem*, 5 (50%) autopsy lungs showed co-infections. Only 2 of these 5 patients with bacterial co-infections had received empirical antibiotics, further supporting the beneficial effect of antibiotics during SARS-CoV-2 infection (Figure 4C). Taken together, bacterial co-infection in lungs was found in 7 (25%) of all 28 *post mortem* SARS-CoV-2 positive cases. Co-infections were not more frequent than in SARS-CoV-2 negative autopsy cases (*n* = 11, 32%) where only 3 out of 11 patients had received antibiotics. This suggests that SARS-CoV-2 infection of the lungs did not lead to a strong predisposition toward co-infections (Figure 4A). Altogether, 75% of SARS-CoV-2 positive cases died without known bacterial co-infection in the lung or end-stage tumors, yet overwhelmingly often of respiratory failure (75%). This is consistent with the interpretation that the majority of SARS-CoV-2 related deaths occurred due to SARS-CoV-2 itself and not due to a co-infection.

The presence of *Staphylococcus aureus* in the lungs correlated with the grade of neutrophilic infiltration, and *Staphylococcus aureus* was more frequently detected in the lungs of patients with signs of acute bronchopneumonia (*p* = 0.0197, Figure 2 and Supplementary Figure 1A). However, the presence of *Staphylococcus aureus* in the lung did not track with the presence of SARS-CoV-2, further strengthening the notion that SARS-CoV-2 does not lead to increased susceptibility toward bacterial co-infections (Supplementary Figure 1B). Time between death and autopsy did not correlate with the amount of *Staphylococcus aureus* detected which makes an influence of *post mortem* time on the detection of respiratory pathogens unlikely (Supplementary Figure 1C).

The duration of hospitalization and of the stay in an intensive care unit (ICU) as well as mechanical ventilation did not correlate with bacterial co-infection in our cohort, most likely because all SARS-CoV-2 positive and negative patients received empirical antibiotics when they were admitted to the ICU and/or mechanically ventilated (Figure 4D). In line with this finding, most patients with a longer stay in hospital were treated with antibiotics which prevented bacterial co-infection (Figure 4D).

When comparing the histomorphological appearance of autopsy lungs of SARS-CoV-2 positive patients without (*n* = 21) and with (*n* = 7) bacterial co-infections, we found that 13 (63%) of the patients with SARS-CoV-2 as the sole pathogen showed histological signs of DAD, mainly in the exudative and/or proliferative stage, whereas none of the patients with co-infections showed DAD (Supplementary Table 5). As expected, SARS-CoV-2 positive patients with bacterial co-infections showed significant neutrophilic infiltration (grade 2 or 3) as a sign of acute bronchopneumonia slightly more frequently [i.e., 4 of 7 (57%) patients with bacterial co-infections, and 9 of 21 (43%) patients without bacterial co-infections]. Intraalveolar hemorrhage and intraalveolar edema were mainly observed in SARS-CoV-2 positive patients without bacterial co-infections (Supplementary Table 5).

Our TaqMan array card detected *Pneumocystis jirovecii* (*n* = 6, 9.7%) and EBV (*n* = 2, 3.2%) in a few patients, yet the qPCR signals in these cases were 64–512× lower than in control cases with clinically relevant *Pneumocystis jirovecii* and EBV infections, indicating very low amounts of these typical fungal and viral pathogens, respectively (Figure 4B). These results were validated either by negative immunohistochemistry using an antibody against Pneumocystis jiroveci or by negative *in situ* hybridization for EBV, suggesting no clinical relevance. In addition, histology did not show typical morphological patterns of pneumocystis pneumonia (interstitial inflammation, intraalveolar frothy eosinophilic exudate, and typical “cysts”) or EBV pneumonia (lymphoid infiltrates) in any of these eight patients.

**FIGURE 5:**
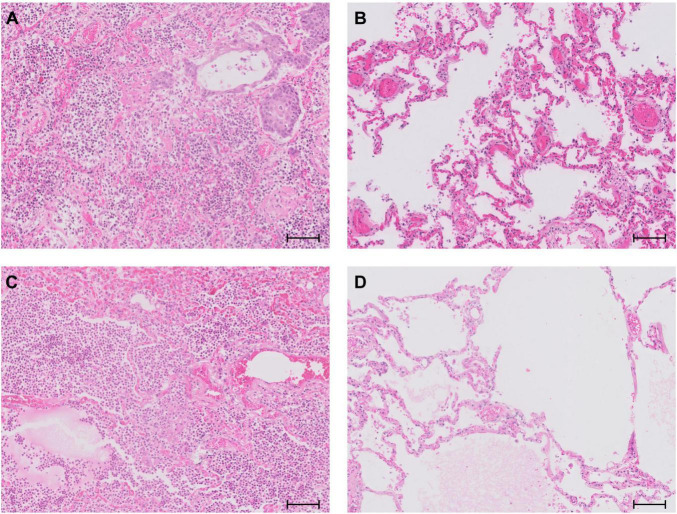
Representative lung histology of **(A)** a patient with clinically known COVID-19 showing DAD with hyaline membranes, heavy inflammatory infiltration and squamous metaplasia; **(B)** a patient with clinically known COVID-19 showing only discrete morphological changes in his lungs; **(C)** a patient who was unexpectedly SARS-CoV-2 positive *post mortem* with bacterial co-infection and signs of acute bronchopneumonia; **(D)** a patient who was unexpectedly SARS-CoV-2 positive *post mortem* with emphysema and discrete edema, but no relevant inflammation. Scale bar, 100 μm.

According to our previous study describing the histopathological characteristics of COVID-19 patients, patients with a clinically known COVID-19 diagnosis prior to death fell into two groups: they showed either massive lung damage with heavy inflammatory infiltration (Figure 5A) or only mild morphological changes (Figure 5B) (24). When compared to COVID-19 autopsy patients identified by *post mortem* analyses of lung tissues, we saw a striking difference of the histomorphological appearance of the autopsy lungs: None of the patients who were unexpectedly SARS-CoV-2 positive showed DAD, only half of these patients showed relevant neutrophilic infiltration (Figure 5C), and the other half did not show COVID-19 typical lung pathology but rather unspecific and common morphological changes in their lung tissues such as emphysema (Figure 5D). Accordingly, the vast majority of clinically known COVID-19 patients (*n* = 17, 94%) died of respiratory failure, whereas only 4 (40%) out of 10 *post mortem* SARS-CoV-2 positive patients died of respiratory failure.

While we did not find other obvious differences in medical histories between autopsy patients with a COVID-19 diagnosis *ante mortem* and those that we identified *post mortem*, patients with a known COVID-19 diagnosis prior to death showed significantly more COVID-19 typical symptoms that were reported upon hospital admission (3.1 on average) than patients who were only diagnosed with COVID-19 *post mortem* (1.4 on average). This might reflect a bias in reporting in COVID-19 patients who have been monitored clinically with particular attention.

When we compared autopsy patients of the first and second pandemic wave, we found more bacterial co-infections among COVID-19 patients during the second wave [in 5 of 14 (36%) SARS-CoV-2 positive patients] compared to the first wave [in 2 of 14 (14%) SARS-CoV-2 positive patients]. This might reflect decreased empirical use of antibiotics during the second wave, as the pandemic progressed in Switzerland, potentially because of later hospital or ICU admission of COVID-19 patients and increased awareness for the risk of multi-drug resistant infections (26, 27). Indeed, among SARS-CoV-2 positive patients of our study, fewer patients received antibacterial treatment during the second wave (57%), compared to the first wave (79%) (Table 1). No differences in histopathological characteristics and medical histories were found between the first and second pandemic waves, with one exception: Two cases of fibrotic DAD were observed during the second pandemic wave and none in patients of the first wave. However, the number of subjects studied is too small to draw a firm conclusion about DAD, and further studies are needed to elucidate potential differences.

Taken together, our autopsy study demonstrates significant underreporting of SARS-CoV-2 infection and COVID-19 deaths in Switzerland. We did not find a higher incidence of respiratory bacterial co-infections in deceased COVID-19 patients, compared to non-COVID-19 control patients who underwent autopsy during the same time periods (first and second pandemic wave in Switzerland). Overall, our data suggest that most COVID-19 patients in Switzerland died from and not with COVID-19.

## Discussion

In this retrospective study, we analyzed a large cohort of consecutive autopsies during the first and second wave of the COVID-19 pandemic in Switzerland. The novel coronavirus SARS-CoV-2 was detected in lung tissues of 28 (45%) out of 62 deceased patients during this period, although only 18 (29%) patients were known to have COVID-19 at the time of autopsy. Of the 10 additional patients, 5 had shown COVID-19 typical symptoms and/or COVID-19 typical autopsy findings. Four of these 5 patients died from respiratory failure and one from a thrombotic coronary artery event, all consistent with a COVID-19 diagnosis (29–31, 32–35). Therefore the likely cause of death in these 5 patients was COVID-19. Thus, our results indicate a 16% higher SARS-CoV-2 infection rate and an 8% higher COVID-19 death rate than officially reported. This result supports the idea that reliable pathogen detection in patients requires tissue testing (36).

Several reasons may have contributed to the high number of unreported COVID-19 cases. Viral copy numbers in the nose may have been too low for detection by swab testing at certain stages of infection, and swabs have a high variation related to the sampled area (37–41). For example, in our cohort, 2 of 10 unexpected SARS-CoV-2 positive patients showed a negative nasopharyngeal swab test within 24 h prior to death (one during the first and one during the second wave of the pandemic). Another reason may have been an atypical or asymptomatic course of infection. Accordingly, nasopharyngeal swab testing was never performed in 8 out of 10 unexpected SARS-CoV-2 positive patients of our cohort (23, 42). A third reason may have been insufficient testing capacities which was an issue in several countries at the beginning of the pandemic such that some patients may have died from COVID-19 and its complications before appropriate diagnosis (3, 22, 43–45). Consistent with that idea, our data identify 31% (5 of 16) non-COVID-19 cases as SARS-CoV-2 positive *post mortem* during the first wave of the pandemic, and only 18% (5 of 28) during the second wave. Fourth, several analytical vulnerabilities of SARS-CoV-2 diagnostic RT-PCR have been suggested as potential causes of false negative results. These include inadequately trained staff which may lead to errors during sampling and sample processing (46, 47). Finally, some unexpectedly SARS-CoV-2 positive patients may have contracted the virus in the hospital and may have died before showing typical symptoms. As a consequence, these patients may not have been tested for the virus. This explanation highlights the possibility of nosocomial SARS-CoV-2 spread and calls for enhanced testing strategies in hospitals (48). Overall, patients with a positive SARS-CoV-2 test upon hospital admission showed more COVID-19 typical symptoms on average, both during the first and during the second wave, compared to patients that we identified as SARS-CoV-2 positive *post mortem*. This may also indicate a bias toward reporting symptoms of known infected patients who have been monitored clinically with particular attention (49, 50). It is of note that patients with a known SARS-CoV-2 infection during the first wave showed more typical symptoms than during the second wave (51). This may have to do with the fact that patients may have been monitored more closely while the infection had still been new to clinicians (51, 52).

Our data need to be put in perspective to the incidence of COVID-19 and mortality data during the first and second pandemic waves, during which public health scenarios and patient management differed substantially in Switzerland (25–28). The first wave of COVID-19 in Switzerland saw implementation of strict public health measures, while limited information on the course of the disease was available. During the second wave, testing capacities had expanded and public health measures were implemented slowly, yet patient management in hospitals had improved and dexamethasone had become standard of care. As a likely consequence, the second wave in Switzerland led to a 13-fold higher number of reported cases (4,00,159 vs. 30,460) yet only to a 5.7-fold higher number of COVID-19 deaths in absolute numbers (5,077 vs. 894). This means that the COVID-19 associated mortality was significantly lower during the second wave (27, 28, 53). Of note, total mortality in Switzerland increased by about a factor of 10 during both waves compared to spring or fall 2018, as a likely consequence of COVID-19, while vaccinations were not available (27). Our data are consistent with better patient management and more reliable testing during the second wave, because a lower percentage of patients was unexpectedly SARS-CoV-2 positive *post mortem* after the first wave. Our data also suggest better familiarity with key symptoms of COVID-19 during the second wave, because the average number of recorded symptoms per patient was higher during the first wave. Surprisingly, in our cohort COVID-19 patients had more bacterial co-infections during the second wave and were treated with antibiotics less frequently than during the first wave, potentially because of later hospital or ICU admission of patients during the second wave in Switzerland (27).

Underreporting cases and underestimation of mortality is common for infectious diseases. Therefore it is not surprising that this was also found during the COVID-19 pandemic (44, 54). COVID-19 related mortality differed across health systems globally, and it has been proposed that underreporting of COVID-19 mortality is most pronounced in developing countries (55, 56), possibly due to limited access to testing or reporting (44, 57). Excess general mortality also differs between countries (45, 58), and even in the United States, approximately 20% of excess deaths in 2020 were not reflected in COVID-19 death counts, suggesting imprecise cause-of-death attribution during the pandemic (59, 60). In this context, it is interesting to mention that mortality data is accessible for many countries, but few countries keep continuous death records for longer time periods. One study is of particular interest as it compares the excess mortality impact of the COVID-19 pandemic in 2020 in Switzerland to Sweden and Spain – the three European countries that have reliable continuous data on death counts which were not impacted by world wars (61). For 2020, all three countries showed the second largest infection-related mortality since the beginning of the 20th century. This study is consistent with our findings in that it finds that excess mortality substantially exceeds official deaths reported from COVID-19 (61).

Bacterial co-infections occurred in SARS-CoV-2 infected patients, yet not more frequently than in other critically ill and hospitalized patients [25% (7/28) in SARS-CoV-2 infected vs. 32% (11/34) in SARS-CoV-2 free patients in our cohort]. However, it has to be borne in mind that many patients in our cohort received antibiotics. Strikingly, only 17% of all patients with an antibiotic coverage had a bacterial co-infection, compared to 46% of patients who did not receive antibacterial treatment. For COVID-19 patients, the protective effect of antibiotics is even more striking. Of all 18 patients with a clinically known COVID-19 diagnosis, 16 received empirical antibiotics, and none of them contracted a bacterial co-infection, not even the 4 mechanically ventilated ones, although mechanical ventilation is a risk factor for bacterial co-infections (62, 63). This is in line with recent data reporting a relatively low incidence of bacterial pulmonary co-infections in critically ill, antibiotic treated COVID-19 patients (20, 64, 65). However, our *post mortem* results are of critical importance as clinical criteria do not reliably predict bacterial infection and they strongly suggest the use of empirical antibiotics in COVID-19 patients (66–68).

The reported incidence of pulmonary fungal co-infections in hospitalized COVID-19 patients is lower than bacterial co-infections (20, 63, 65, 69). In line with these data, we did not detect fungal species in SARS-CoV-2 positive patients of our cohort by histological stains for fungi or by immunohistochemistry for *Pneumocystis jirovecii*. However, a more recent study describes co-infections with different fungal species as a relevant finding after long-term treatment of COVID-19 patients (20, 63, 69). The fact that only 2 out of 18 clinically known COVID-19 patients stayed in hospital for >2 weeks (17 and 30 days, respectively) may at least partially explain the lack of fungal co-infections in our autopsy specimens (69).

The pathology of severe COVID-19 involved profound dysregulation of the immune system, which may change the susceptibility of patients to bacterial co-infections (70). Several mechanisms may account for that. First, it has been observed that SARS-CoV-2 leads to profound structural alterations of lymph nodes and spleens (33, 71). As the structure of secondary lymphoid organs is essential for immune defense against microbes, their breakdown during an ongoing virus infection may temporarily reduce immune competence toward other infections (72). Second, it has been suggested that COVID-19 associated lymphopenia may contribute to a higher rate of bacterial co-infections (73, 74). Finally, signs of T cell exhaustion, such as PD-L1 overexpression, are observed in some COVID-19 patients, and this may further limit the immune competence of COVID-19 patients toward other pathogens (75–78). Since multiple risk factors for bacterial co-infections have been described in COVID-19 patients, it is likely that multiple immunological mechanisms are at play and determine the individual risk (74).

Our autopsy findings lead us to conclude that in 82% (23/28) of SARS-CoV-2 infected patients, and in 37% (23/62) of all cases the cause of death was related to SARS-CoV-2 infection. In 75% (21/28) of all SARS-CoV-2 positive patients the cause of death was respiratory failure due to DAD and/or pneumonia, including 4 unexpectedly SARS-CoV-2 positive patients showing COVID-19 typical inflammatory infiltrates and signs of acute bronchopneumonia upon autopsy. Since only 29% (18/62) of all autopsy cases were reported as clinical COVID-19 cases, our study reveals an 8% higher SARS-CoV-2 related mortality rate than officially reported. Thus, the widely discussed excess all-cause mortality in the years 2020/21 is likely insufficiently explained through officially reported COVID-19 deaths and may include a high number of unreported cases (79–81).

## Limitations

Limitations of our study include the size of the autopsy cohort. Due to the relatively low number of individuals, the role of distinct medications used in each period could not be assessed (e.g., corticoids, hydrochloroquine, remdesivir, and tocilizumab). A second limitation is the size of the analyzed tissue samples, which might not be representative for the entire organ. We addressed this limiting factor by taking at least two representative tissue blocks from different lobes of both lungs. The clinical symptoms that we analyzed as “COVID-19 typical symptoms” are not specific for COVID-19, i.e., they do not allow to firmly exclude other diseases, especially other less common infections that were not included on our TaqMan respiratory array. Further studies are needed to elucidate this question in more detail. In addition, we did not distinguish different SARS-CoV-2 strains.

A further caveat is that the conclusions from our study may not be generalizable to all countries. There is wide variation in reporting COVID-19 deaths and in the use of antibiotics between different health systems (44, 82–85). However, as rules for COVID-19 reporting in Switzerland are stringent, it is likely that underreporting of COVID-19 mortality is even more pronounced in other countries.

## Conclusion

Our study not only shows a high number of unreported COVID-19 cases, but also a higher number of COVID-19 related deaths than reported, supporting the hypothesis that the SARS-CoV-2 infection rate in the population is underestimated and that COVID-19 is responsible for the unexplained number of excess mortality observed since 2020. Furthermore, our study emphasizes the value of autopsies to study pathogenesis and epidemiological aspects of infectious diseases. *Post mortem* testing during the pandemic allows to better track the presence of the virus in the population. According to our data, most COVID-19 patients die from and not with the disease: The majority of SARS-CoV-2 positive patients died from respiratory failure, while co-infections played a minor role in fatal COVID-19.

## Data Availability Statement

The original contributions presented in the study are included in the article/Supplementary Material, further inquiries can be directed to the corresponding author.

## Ethics Statement

The studies involving human participants were reviewed and approved. This study was conducted according to the principles expressed in the Declaration of Helsinki. Ethics approval was obtained in written form from the Ethics Committee of Northwestern and Central Switzerland (Project-ID 2020-00629). For all patients, personal family consent was obtained for the autopsy and sample collection, in line with the Swiss law and the Ethics approval. The patients/participants provided their written informed consent to participate in this study.

## Author Contributions

NS, RN, NW, TJ, and KM conceived the ideas and design. NS, RN, MH, AB, AG, CH, VZ, NW, and KM performed the data collection. NS, RN, AG, AF, NM, JK, CH, MS, TD, VZ, and KM performed the experiments, data analysis, and interpretation. NS, RN, MH, NW, TJ, and KM did the manuscript drafting and editing. All authors contributed to the article and approved the submitted version.

## Conflict of Interest

TJ is an employee of Novartis Pharma AG. The remaining authors declare that the research was conducted in the absence of any commercial or financial relationships that could be construed as a potential conflict of interest.

## Publisher’s Note

All claims expressed in this article are solely those of the authors and do not necessarily represent those of their affiliated organizations, or those of the publisher, the editors and the reviewers. Any product that may be evaluated in this article, or claim that may be made by its manufacturer, is not guaranteed or endorsed by the publisher.
