# Supplementary figures and images for: COVID-19 Autopsies Reveal Underreporting of SARS-CoV-2 Infection and Scarcity of Co-infections
Source: Front Med (Lausanne). 2022 Apr 14;9:868954. doi: 10.3389/fmed.2022.868954 (PMC9046787; doi:10.3389/fmed.2022.868954)

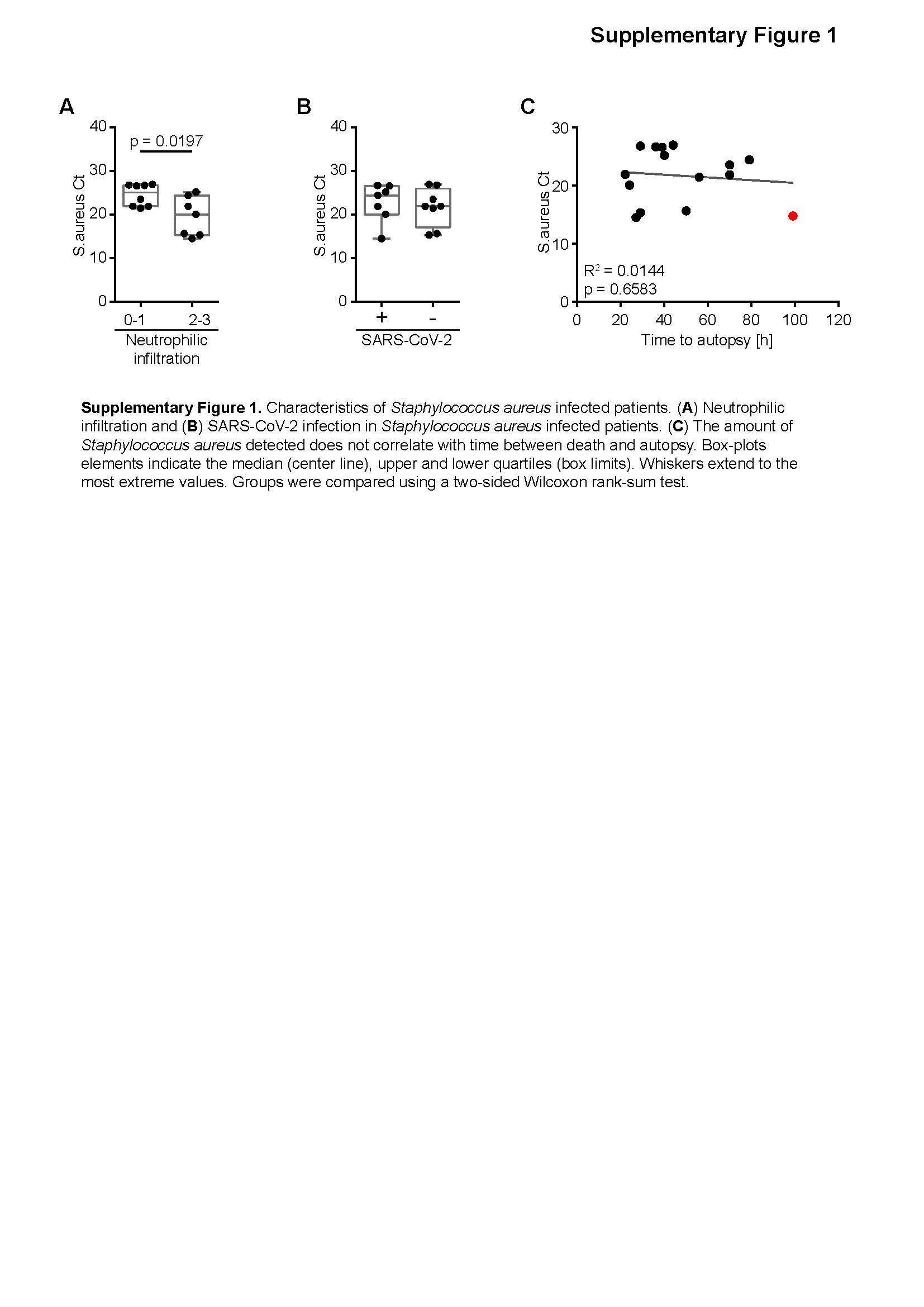

Supplement: Supplementary file 2 [file Image_1.jpg]
